# Influence of arbuscular mycorrhizal colonization on whole‐plant respiration and thermal acclimation of tropical tree seedlings

**DOI:** 10.1002/ece3.1952

**Published:** 2016-01-18

**Authors:** Catherine Fahey, Klaus Winter, Martijn Slot, Kaoru Kitajima

**Affiliations:** ^1^ Department of BiologyUniversity of FloridaGainesvilleFlorida32611; ^2^ Smithsonian Tropical Research InstituteApartado 0843– 03092BalboaAncónRepublic of Panama; ^3^ Graduate School of AgricultureKyoto UniversityKyoto606‐8502Japan

**Keywords:** Acclimation, carbon, climate, Panama, phosphorus, symbiosis

## Abstract

Symbiotic arbuscular mycorrhizal fungi (AMF) are ubiquitous in tropical forests. AMF play a role in the forest carbon cycle because they can increase nutrient acquisition and biomass of host plants, but also incur a carbon cost to the plant. Through their interactions with their host plants they have the potential to affect how plants respond to environmental perturbation such as global warming. Our objective was to experimentally determine how plant respiration rates and responses to warmer environment are affected by AMF colonization in seedlings of five tropical tree species at the whole plant level. We evaluated the interaction between AMF colonization and temperature on plant respiration against four possible outcomes; acclimation does or does not occur regardless of AMF, or AMF can increase or decrease respiratory acclimation. Seedlings were inoculated with AMF spores or sterilized inoculum and grown at ambient or elevated nighttime temperature. We measured whole plant and belowground respiration rates, as well as plant growth and biomass allocation. There was an overall increase in whole plant, root, and shoot respiration rate with AMF colonization, whereas temperature acclimation varied among species, showing support for three of the four possible responses. The influence of AMF colonization on growth and allocation also varied among plant species. This study shows that the effect of AMF colonization on acclimation differs among plant species. Given the cosmopolitan nature of AMF and the importance of plant acclimation for predicting climate feedbacks a better understanding of the patterns and mechanisms of acclimation is essential for improving predictions of how climate warming may influence vegetation feedbacks.

## Introduction

Tropical forests are a major component of the global carbon cycle as they account for more than a third of terrestrial net primary productivity (Beer et al. 2010). Therefore, differential effects of climate factors on photosynthesis and respiration rate in these ecosystems could shift the global carbon balance in response to future climate change. The effect of increasing temperatures on autotrophic dark respiration has been an important area of study in recent years because of the need to improve coupled climate‐vegetation models. Recent studies highlight the potential importance of temperature acclimation (Wythers et al. 2005; King et al. 2006; Atkin et al. 2009; Slot et al. 2014; Vanderwel et al. 2015). Thermal acclimation is a biochemical, physiological, or structural adjustment by an individual plant in response to a change in the temperature regime that results in a shift in the short‐term response to temperature. Acclimation to warming commonly results in down‐regulation of respiration rates at a given temperature (Slot and Kitajima 2015), such that acclimated plants experience lower respiratory carbon loss at their new temperature than nonacclimated plants (Atkin and Tjoelker 2003). Our understanding of respiratory responses remains limited for tropical species, particularly at the whole‐plant level. Therefore, gaining a better understanding of the factors that influence respiratory acclimation in tropical forests should be a priority given their potential influence on global carbon cycle. Our study focused on increased nighttime temperature because historical warming trends are asymmetric (Vose et al. 2005; IPCC, 2007; Lobell et al. 2007) and because of the importance of nighttime temperature for tropical forest growth and the hypothesized role of nighttime respiration (Clark et al. 2010, 2013).

Belowground processes including activities of decomposers and root symbionts contribute to more than half of ecosystem respiration from tropical forests (Chambers et al. 2004; Malhi 2012). Whole‐plant respiration measurements give us a more comprehensive understanding of metabolic activity because different plant parts show different responses to temperature (Loveys et al. 2003) and roots and associated symbionts can respond differently to environmental change (Alberton et al. 2005; Vega‐Frutis et al. 2014). Arbuscular mycorrhizal fungi (AMF) in the phylum Glomeromycota that form symbiotic associations with plants are ubiquitous in tropical forests (Alexander and Lee 2005). These fungi can act as an extension of the root, providing access to additional mineral nutrients, especially phosphorus, in exchange for assimilated carbon from the plant. Although AMF may improve nutrient acquisition and net carbon uptake by host plants, they are also substantial sinks for as much as 20% of assimilated carbon (Pearson and Jakobsen 1993; Jakobsen et al. 2002).

Arbuscular mycorrhizal fungi colonization may influence the whole‐plant respiratory carbon flux through increased demand for energy for growth and metabolism because of enhanced nutrient uptake. Additionally, rapid turnover of fungal structures and cellular costs of colonization have the potential to increase root respiration (Bago et al. 2003; Staddon et al. 2003). AM roots typically exhibit higher rates of respiration than non‐AMF roots (Baas et al. 1989; Valentine and Kleinert 2007; Atkin et al. 2009), however, it is unknown how AMF colonization affects respiration of aboveground portions of tropical plants. Fungal hyphae themselves have additional respiratory costs associated with nutrient translocation, growth, and maintenance. Respiration by extraradical AMF hyphae associated with tropical trees accounts for greater than one‐third of root respiration (Nottingham et al. 2010). Because the majority of studies on temperature response of plants have been conducted under controlled conditions with unknown mycorrhizal status, empirical data are needed on how AMF colonization affects plant respiratory response to growth temperature.

AMF may not only influence respiration rate of host roots, but also the thermal acclimation of plants. Atkin et al. (2009) showed that *Plantago lanceolata* roots colonized by AMF had decreased capacity for long‐term acclimation to cold temperatures. In contrast, extraradical fungal mycelium of AMF associated with the same species acclimated rapidly to heating (Heinemeyer et al. 2006). Thus, AMF may stimulate root respiration, but constrain acclimation of root respiration to low temperature, whereas the fungus itself appears to have the capacity for acclimation. The overall effect of AMF on whole‐plant respiration in response to warming remains largely unknown. Given the highly variable short‐term temperature responses of leaf respiration across tropical tree species (Slot et al. 2013), it is likely that species also differ in how whole plant respiration is influenced by AMF colonization and long‐term temperature change.

**Figure 1:**
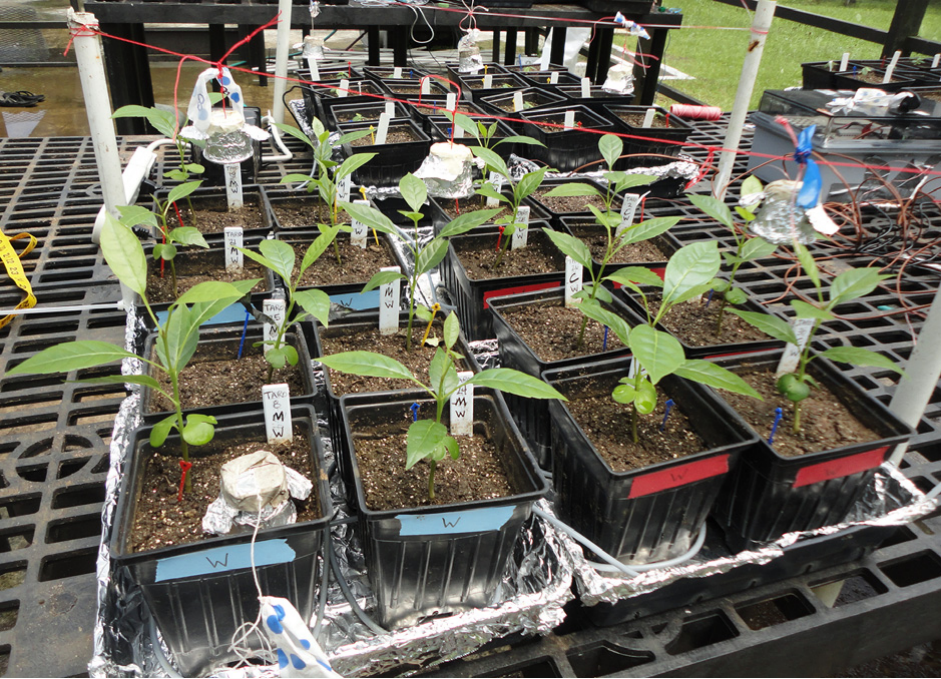
Seedlings in greenhouse with experimental nighttime warming treatment.

**Figure 2:**
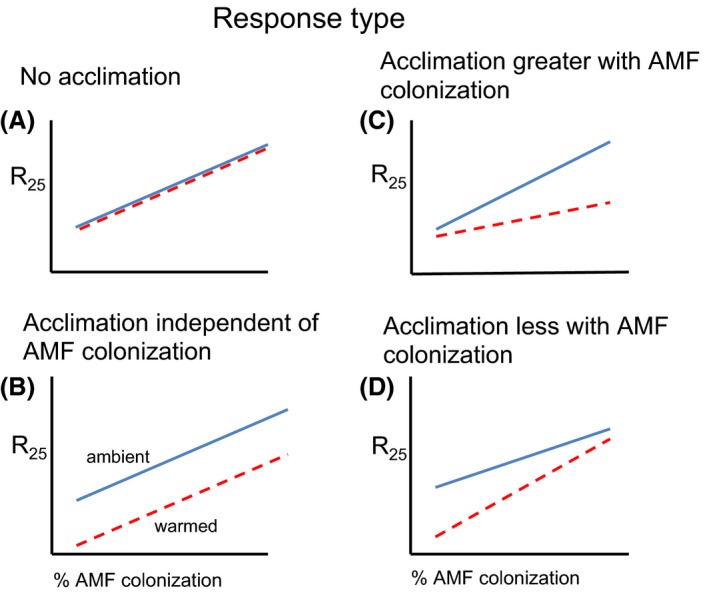
Graphical illustrations of four possible temperature acclimation responses of plant dark respiration measured at 25°C (R_25_). A) no acclimation regardless of mycorrhizal colonization; B) acclimation independent of mycorrhizal colonization; C) greater acclimation with mycorrhizal colonization; and D) Less acclimation with mycorrhizal colonization. All cases assume increased respiration rate with AMF colonization.

The goals of this study were to assess (1) how respiration rates of above‐ and belowground plant parts of tropical tree seedlings change with mycorrhizal colonization; (2) the effect of AMF colonization on acclimation of respiration to increased nighttime temperature; and (3) how seedling growth rate and carbon allocation vary with AMF colonization and nighttime temperature. To address these questions we grew seedlings of five tree species in sterilized soils with and without inoculation with AMF spores in a shade house in Panama, and subjected them to 1–12 week treatments of elevated and ambient nighttime temperature (Fig. 1). We tested how respiration rate and its thermal acclimation would be influenced by AMF colonization with four possible scenarios (Fig. 2), where we hypothesized that AMF colonization would increase whole‐plant respiration. In scenario A, if respiration rates do not acclimate to growth temperature, we would see no difference in respiration rate between the temperature treatments and the AMF‐respiration response curves would overlap (Fig. 2). If acclimation is independent of mycorrhizal status, we would expect a parallel downward shift of the relationship for warm‐acclimated plants compared to ambient grown plants, but no difference in slope of these two lines (Scenario B, Fig. 2). If AMF colonization stimulates, or impedes acclimation then we would, respectively, see a decrease (Scenario C, ‘AMF‐stimulated acclimation’) or an increase (Scenario D, ‘AMF‐suppressed acclimation’) in slope of the warmed lines relative to those of control plants at ambient temperature (Fig. 2).

## Methods

### Seedling growth conditions

Seeds of *Ficus insipida* Willd., *Ochroma pyramidale* (Cav. ex Lam.) Urb*., Luehea seemanii* Triana & Planch, *Castilla elastica* (Liebm.) C.C. Berg, and *Tabebuia rosea* DC were collected in secondary forests near Gamboa, Panama, and in Parque Natural Metropolitano, a 120‐year‐old secondary forest on the Pacific side of Panama. Henceforth, species will be referred to by their genus name only. Seeds were surface sterilized by soaking in 0.6% sodium hypochlorite (Clorox Company, Oakland, CA) for 10 min and rinsing twice for 10 min in DI water. Seeds of *Ochroma* were placed in 80°C water for 3 min prior to sterilization to release dormancy. Seeds were then spread evenly across the surface of germination trays filled with a 1:1 mixture of river sand and vermiculite, except *Ficus* seeds, which were germinated on 3:1 sand to vermiculite. Sand and vermiculite were autoclaved for 1 h at 121°C. All tools and pots were sterilized in 0.6% sodium hypochlorite for 24 h before use. Germination and initial seedling growth prior to experimental treatment were conducted in a screened enclosure built on a concrete pad in the Santa Cruz Experimental Field Facility in Gamboa, Panama. Seedlings received approximately 30% full sun, and were watered to field capacity every morning. After germination, they were transplanted into sterile 4:1:1 soil: vermiculite: river sand mix in pots (1 L). The sterile growth mixture was prepared by autoclaving two times for 1 h at 121°C, separated by 12–24 h (Wolf et al. 1989). *Castilla* and *Ficus* seedlings were transplanted individually to pots, whereas *Luehea, Ochroma*, and *Tabebuia* seedlings were transplanted with three seedlings per pot (1 L), later reduced to one seedling per pot, selecting similar seedling size across pots. Thirty‐two seedlings of each species were dried and weighed to determine the pretreatment biomass for calculation of relative growth rate. Half of the seedlings of each species (20 seedlings) were randomly selected for experimental heating and half of the seedlings in each temperature treatment were inoculated with AMF (See Table 1 for final sample sizes). Treatments were initiated once seedlings began to produce their first true leaf.

**Table 1:** Sample sizes (number per treatment), percent showing nonzero colonization (of the number of seedlings inoculated with AMF), mean percent root length colonized for seedlings with nonzero colonization, seedling age at harvest, duration of the warming treatment, average final seedling mass, and average seed mass of five neotropical tree species

	*Ficus*	*Luehea*	*Ochroma*	*Tabebuia*	*Castilla*
Sample size in ambient treatment	19	11	10	15	18
Sample size in warming treatment	16	13	9	16	15
Percent of inoculated seedlings colonized (*n*)	70.6 (17)	86.7 (15)	100 (12)	43.8 (16)	0 (17)
Percent root length colonized (± SE)	32.6 (4.8)	36.0 (6.6)	46.4 (7.9)	46.4 (11.7)	n/a
Age at harvest (days)	70–94	78–126	44–110	47–110	33–103
Mean length of warming in days (range)	46 (30–61)	72 (42–90)	62 (9–76)	53 (24–87)	20 (6–74)
Mean plant mass (g)	0.56	0.65	1.29	0.66	1.54
Mean seed mass (g)	0.001	0.002	0.007	0.03	0.5

### Experimental setup

Soil samples were collected in a secondary forest near Gamboa, Panama, from under adult trees of the five species, pooled, and thoroughly mixed. Mycorrhizal spores were extracted from soil by wet sieving through a 38 *μ*m sieve followed by sucrose centrifugation (Daniels and Skipper 1982). AM spores were applied as a liquid wash at the base of each plant and covered with a thin layer of soil. Nonmycorrhizal plants received autoclave‐sterilized inoculum and a filtrate rinse. Additionally, four pots with soil, sterile inoculum, and filtrate but no plants were used to calculate the amount of nonmycorrhizal microbial respiration. These pots were watered along with the experimental pots.

Two types of nighttime warming setup were used because of space constraints. In the first type, potted seedlings of *Luehea*,* Tabebuia*, and *Ficus* were placed in plastic trays (28 × 28 × 4 cm) lined with aluminum foil (4 pots per tray). Flexible heat rope (Big Apple Herpetological, Inc., New York) was placed across the bottom of the trays around each pot and connected to a thermostat set to trigger warming when temperature dropped below 27°C. This resulted in an average nighttime warming of 3.3°C above ambient air temperature (Table S1). In the second warming setup, *Castilla* and *Ochroma* seedlings were placed in clear plastic open top chambers (volume of 2.55 m^3^). In the warming treatment the temperature of the incoming air was elevated by 3°C with resistance heaters from 6 pm to 6am (See Cheesman and Winter (2013a) for detail on this setup). The differences in warming set up reduce our ability to compare *Castilla* and *Ochroma* responses to the other species.

Temperature was monitored throughout the experiment with iButton data loggers (Maxim Integrated Products, Sunnyvale, CA) placed on the soil surface and suspended near the seedling leaves (Figure S1). These measurements showed similar ambient and warmed temperature regimes between the two types of heating treatments (Figure S2). Within the temperature treatments, pots were randomly rearranged on a weekly basis for the duration of the experiment.

### Respiration measurements

Whole‐plant respiration was measured when seedlings were 10–20 cm tall. Plants could not all be measured at the same age because the measurements for each plant took on average 4 h, so measurements took several months to complete. Because of growth rate differences across species, the length of warming treatment varied among plants (ranging from 1 to 12 weeks), but on average 98%, 97%, 97%, and 66% of biomass developed under the experimental treatments (for *Ficus, Luehea, Ochroma,* and *Tabebuia* respectively). Plants were kept in the dark for 2–6 h, and watered to field capacity before measurements. Gravimetric soil moisture was measured after respiration measurements were completed and average soil moisture at the time of measurement was 58.9 ± 7.4% and 58.8 ± 7.1% (SD) for the ambient and warmed pot respectively. Respiration rate at 25°C (R_25_) was measured with one of two custom‐built whole‐plant respiration chambers of two sizes (27 and 4.7 L) connected to an open‐flow gas‐exchange system consisting of Walz components (Walz, Effeltrich, Germany) and an infrared gas analyser (LI‐6252; Licor, Lincoln, NE). The smaller chamber was used for small plants that would take too long to equilibrate in the large chamber. We confirmed that the same plants measured in both chambers produced comparable R_25_. The temperature of the chambers was maintained at 25°C by placing them inside a temperature‐controlled growth cabinet (Environmental Growth Chambers, Chagrin Falls, OH). When the flux rate was stable—which took 1–2 h—respiration values were recorded. After respiration of the whole plant was recorded, the shoot was cut at the soil surface and the pot with roots and soil was returned to the respiration chamber to measure belowground respiration, i.e., combined CO_2_ flux from the roots, AMF, and soil. The difference between whole plant respiration and belowground respiration was attributed to shoot respiration. To estimate root + mycorrhizal respiration, nonmycorrhizal soil microbial respiration, which was estimated by measuring pots that contained microbial wash but no plant, was subtracted from the total belowground respiration. This nonmycorrhizal soil microbial respiration averaged 0.0044 *μ*mol CO_2_ sec^−1^ per pot.

After respiration measurements, all seedlings were harvested. Leaf area of each seedling was measured with a leaf area meter (LI‐3000A, Licor). Roots were carefully removed from bulk soil and rinsed with tap water to remove adhering soil. Leaf, root, and stem mass were weighed after drying at 60°C for at least 72 h. A small amount of fresh fine roots from across each root system was stored in 70% ethanol for quantifying percent mycorrhizal colonization. Relative growth rate (RGR) of seedlings was calculated as:

$$\text{RGR}=\frac{\left(\text{In}\;{\text{DM}}_{\mathrm{final}}-\text{In}\;{\text{DM}}_{\mathrm{initial}}\right)}{\left(t_{\mathrm{final}}-t_{\mathrm{initial}}\right)}$$

where DM_final_ is the individual whole plant dry mass at final harvest, *t*_final_, and DM_initial_ is the mean dry mass of the seedlings at the initial harvest, *t*_inital_. The initial harvest was conducted immediately before applying treatments.

### Mycorrhizal colonization assessment

In order to quantify the percentage of root length colonized by AMF, fine root samples were cleared with 10% KOH at 80°C for 15–60 min depending on species. Then, the roots were acidified in 1% HCl for 10 min, stained in acidic glycerol with trypan blue at 80°C for 15 min, and destained in acidic glycerol for at least 8 h. Roots were cut into 1 cm fragments and mounted on microscope slides with acidic glycerol. Percent root length colonized by AMF was estimated with the gridline intersect method (McGonigle et al. 1990).

### Shoot phosphorus

To establish the effect of mycorrhizal colonization on phosphorus nutrition of the seedlings, we measured phosphorus content of seedling stems and leaves. Seedlings with >300 mg of shoot dry mass were measured individually. Seedlings with shoot mass <300 mg were systematically paired within species based on temperature treatment and similarity in mycorrhizal colonization to have sufficient tissue for the *P* analysis. This resulted in a total of 114 samples (28 pairs of seedlings and 86 individual seedlings). Total shoot *P* was determined from finely ground samples by combustion in a muffle furnace (>4 h at 500°C) and dissolution of the ash in 50 mL of 1 molL^−1^ H_2_SO_4_. Phosphorus concentration from the ash was determined by automated colorimetry at 420 nm on a Lachat QuikChem 8500 (Hach Ltd., Loveland, CO).

### Statistical analyses

All statistical analyses were carried out in R version 3.2.2 (R Development Core Team 2015). The effects of percent mycorrhizal colonization, and temperature treatment on the dependent variables; R_25_, specific leaf area (SLA; leaf area per unit leaf mass), leaf area ratio (LAR; total leaf area over total plant mass), RGR, and shoot: root ratio; were tested with linear mixed models using the lme function in the nlme package in R with plant age at harvest as a random effect. *Castilla*, which was not colonized by AMF (see below), was analyzed against temperature treatment with a Welch's *t*‐test. Percent AMF colonization for colonized seedlings of each species was tested against temperature treatment and plant age with a linear model. A 2‐way ANOVA was used to test shoot phosphorus concentration against nighttime temperature treatment and AMF colonization as a categorical variable (i.e. colonized or not colonized).

## Results

### AMF colonization

**Figure 3:**
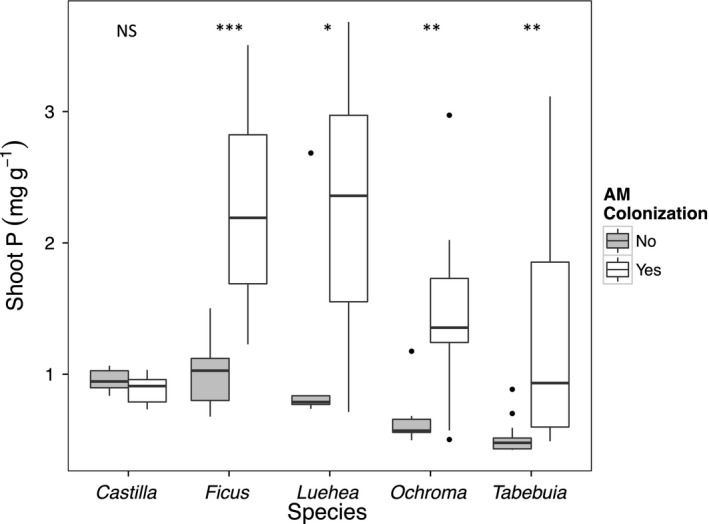
Shoot phosphorus concentration for seedlings of five Neotropical tree species with AM colonization (percent colonization >0, white bars) or without AM colonization (percent colonization = 0, grey bars). In *Castilla* there was no observed AM colonization, but the data were shown separately by AM inoculation treatments in a manner to correspond to other species. Stars indicate differences between mycorrhizal and nonmycorrhizal seedlings within each species (NS:* P* > 0.05, *: *P* < 0.05, **: *P* < 0.01, ***: *P* < 0.001).

The AMF inoculation treatment produced a range in percent AMF colonization in all species except for *Castilla* (Table 1)*. Castilla* seedlings showed no sign of colonization at the end of the experiment, and therefore were excluded from all further analyses that involved AMF colonization. Contamination by AMF in the uninoculated soils was low (less than 8% of uninoculated plants showed colonization). Percent root length colonized for seedlings with nonzero colonization was similar across species, although maximum colonization was lower for *Ficus*. The nighttime warming treatment had no effect on percent AMF colonization (Table S1). Because seedlings were harvested at different ages, we modeled percent colonization for seedlings with nonzero colonization against seedling age. Age affected percent colonization in only one species, *Luehea*, where there was a decrease in colonization with seedling age at harvest (*F*_1,13_ = 8.2, *P* = 0.01). Shoot *P* content increased significantly with mycorrhizal colonization, indicating that percent colonization was a good indicator of the activity of the mycorrhizae (Fig. 3, *P* < 0.05 for all species).

### Respiration and acclimation

**Figure 4:**
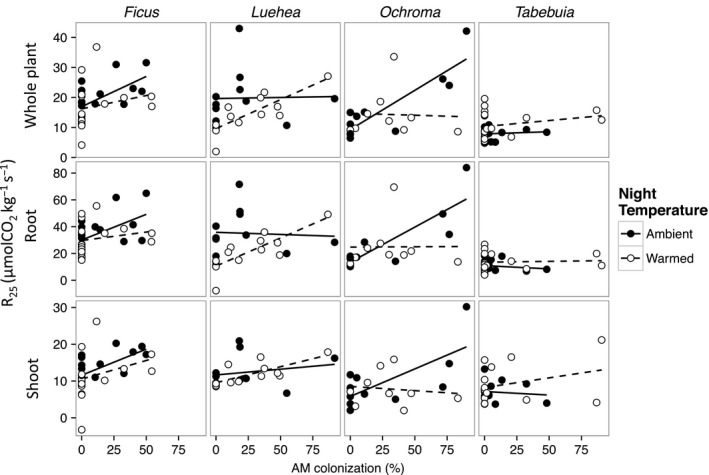
Respiration rate measured at 25°C (R_25_) per unit dry mass of whole plant, root, and shoot in relation to percent colonization by arbuscular mycorrhizal (AM) fungi for ambient and warmed seedlings of four Neotropical tree species. Solid lines indicate the linear regression for ambient grown plants and dashed lines indicate the linear regression for warm grown plants. Each point represents one plant.

Species differed in their responses to the warming treatment and mycorrhizal colonization (Table 2). The positive effect of AMF colonization on respiration was significant in two of the four species (Fig. 4). *Ficus* seedlings showed an increase in whole plant, root, and shoot respiration rate with AMF colonization, but no significant acclimation regardless of AMF colonization, in accordance with Scenario A (Fig. 4). In *Luehea* seedlings, shoot respiration tended to increase with AMF colonization (*P* = 0.06 for the AMF main effect; Fig. 4). *Luehea* showed significant acclimation of root respiration (*P* < 0.05 for the temperature main effect) but only marginal acclimation of whole plant respiration (*P* = 0.08) and no acclimation of shoot respiration (Fig. 4). There was a trend for less acclimation of root respiration with higher AMF colonization for *Luehea*, as in Scenario D (*P* = 0.06 for the temperature × AMF interaction; Fig. 4). Whole plant, root, and shoot respiration rate of *Ochroma* increased significantly with AMF colonization. For whole plant and root respiration, acclimation tended to be greater at high AMF colonization levels in line with Scenario C (*P* = 0.06 and 0.08, respectively, for AMF × temperature interactions; Fig. 4). *Tabebuia* seedlings had no significant response of respiration to AMF colonization and showed no acclimation, equivalent to a flat‐line in Scenario A. *Castilla*, which had no sign of AMF colonization, showed no difference in whole plant, root, or shoot respiration rate between the warmed and ambient treatments, i.e. no acclimation (Figure S3). Root R_25_ was more than twice that of shoot R_25_ per unit dry mass for all species. The ratio of root R_25_ to shoot R_25_ did not differ with percent AMF colonization or across species (Figure S4).

**Table 2:** Linear mixed effects model analysis of the effects of AM colonization (percent root length colonized) and nighttime temperature treatment on respiration rate per unit mass of whole seedlings, shoots, and roots measured at 25°C for four species of neotropical trees. Bold type indicates statistical significance of *P* ≤ 0.05

	df	*Ficus*		*Luehea*		*Ochroma*		*Tabebuia*	
		*F*	*P*	*F*	*P*	*F*	*P*	*F*	*P*
Whole plant									
AM colonization	1	**6.2**	**0.02**	2.6	0.13	**12.7**	**0.01**	1.8	0.21
Night temperature	1	0.9	0.36	3.8	0.08	1.3	0.30	3.2	0.10
AM: temperature	1	0.9	0.35	2.8	0.12	5.4	0.06	0.1	0.78
Shoot									
AM colonization	1	**8.1**	**0.01**	4.5	0.06	**6.2**	**0.047**	2.5	0.14
Night temperature	1	0.9	0.34	0.3	0.57	1.9	0.21	1.8	0.21
AM: temperature	1	0.3	0.61	1.0	0.33	2.4	0.18	0.8	0.38
Root									
AM colonization	1	**5.4**	**0.03**	2.5	0.15	**10.2**	**0.02**	0.1	0.72
Night temperature	1	0.6	0.43	**7.0**	**0.02**	0.9	0.36	2.5	0.14
AM: temperature	1	1.3	0.27	4.5	0.06	4.3	0.08	0.3	0.58

### Growth and morphology

**Figure 5:**
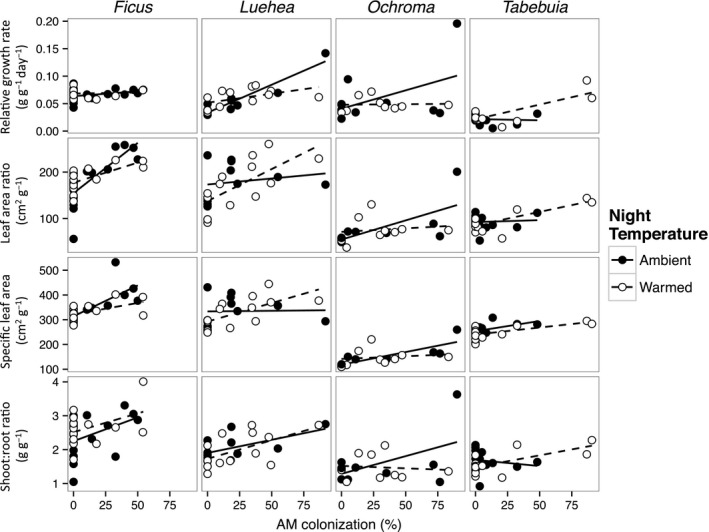
Seedling relative growth rate, leaf area ratio, specific leaf area, and shoot:root ratio as a function of percent root length colonized by arbuscular mycorrhizal (AM) fungi for ambient and warmed seedlings of four Neotropical tree species. Solid lines indicate the linear regression for ambient grown plants and dashed lines indicate the linear regression for warm grown plants. Each point represents one plant.

The effects of AM colonization and nighttime temperature on growth, morphology, and biomass allocation were variable among species (Table 3). RGR was not affected by either of the treatments in *Ficus* and *Ochroma* (Fig. 5). RGR of *Luehea* increased with AMF colonization but to a lesser degree in warmed seedlings (*P* < 0.05 for AMF × temperature interaction). *Tabebuia* also showed increased RGR with AMF colonization (*P* < 0.001 for AMF main effect).

**Table 3:** Linear mixed effects model analysis of the effects of AM colonization (percent root length colonized) and nighttime temperature treatment on specific leaf area (SLA), leaf area ratio (LAR), shoot: root ratio, and relative growth rate (RGR) of seedlings of four Neotropical tree species. Bold type indicates statistical significance of *P* ≤ 0.05

	df	*Ficus*		*Luehea*		*Ochroma*		*Tabebuia*	
		*F*	*P*	*F*	*P*	*F*	*P*	*F*	*P*
RGR									
AM colonization	1	0.02	0.89	**15.0**	**0.003**	1.3	0.29	**31.4**	**<0.001**
Temperature	1	1.3	0.26	0.2	0.64	0.1	0.82	1.5	0.24
AM: Temperature	1	0.1	0.71	**12.3**	**0.005**	0.9	0.38	4.2	0.06
SLA									
AM colonization	1	**27.4**	**<0.001**	2.9	0.12	**6.3**	**0.046**	**12.9**	**0.004**
Temperature	1	0.8	0.37	0.2	0.68	0.5	0.50	**5.3**	**0.04**
AM: Temperature	1	**4.6**	**0.045**	3.0	0.11	1.1	0.33	0.5	0.50
LAR									
AM colonization	1	**35.0**	**<0.001**	**6.5**	**0.03**	1.3	0.29	**15.1**	**0.002**
Temperature	1	1.0	0.33	0.4	0.56	0.04	0.85	0.04	0.85
AM: Temperature	1	**4.9**	**0.04**	3.2	0.10	0.2	0.64	0.4	0.54
SRR									
AM colonization	1	**6.3**	**0.02**	**9.5**	**0.01**	2.7	0.15	4.4	0.06
Temperature	1	1.7	0.21	0.4	0.54	0.04	0.84	1.5	0.24
AM: Temperature	1	0.1	0.77	0.3	0.60	1.7	0.24	2.3	0.16

**Figure 6:**
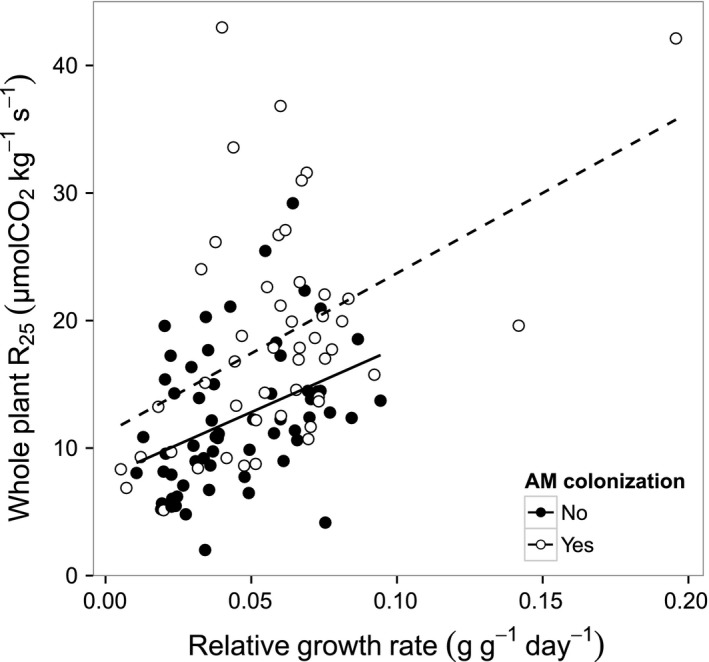
Correlation between whole plant respiration and seedling relative growth rate for seedlings with AM colonization or without AM colonization. The dashed line and the black lines indicate the linear regression for AM colonized seedlings and non‐AM colonized respectively.

Arbuscular mycorrhizal fungi colonization was positively correlated with LAR in *Ficus, Luehea,* and *Tabebuia* and SLA in *Ficus*,* Ochroma*, and *Tabebuia* (Fig. 5). Warming decreased SLA in *Tabebuia,* but the magnitude of this effect was small. In *Ficus*, SLA and LAR increased more strongly with AMF colonization in ambient than in warmed seedlings (*P* = 0.04 for AMF × temperature interaction). *Ficus* and *Luehea* had higher shoot to root ratio with increased AMF colonization and *Tabebuia* showed a trend for this effect but shoot to root ratio was unaffected by the temperature treatment for all species (Fig. 5). Whole plant R_25_ was positively correlated with relative growth rate across species (Fig. 6).

## Discussion

### AMF colonization

Levels of mycorrhizal colonization were similar for all species with the exception of *Castilla,* which showed no signs of colonization in our experiment, even though the species is known to be facultatively mycorrhizal (Janos 1980). *Castilla* had much larger seeds (0.5 g) compared to the other four species (0.001–0.03 g), and the presence of larger seed reserves may have delayed establishment of AMF colonization (Allsopp and Stock 1995; Jin et al. 2009).

Several studies with temperate species have found that temperature affected AM colonization (Heinemeyer and Fitter 2004; Gavito et al. 2005; Hu et al. 2015); however, in the current study, AM colonization was independent of growth temperature, although we cannot rule out the possibility of an increase in extraradical hyphal biomass as we only measured root colonization (Hawkes et al. 2008).

### Respiration and acclimation

The primary goal of this study was to test the potential interactive effects of AMF colonization and warming on respiration, growth, and biomass allocation in tropical tree seedlings. Such interactive effects have been assessed only by one previous study that examined the effects of AMF colonization on cold‐temperature acclimation of root respiration in a temperate forb, *Plantago major* (Atkin et al. 2009). Atkin et al. (2009) showed that root respiration increased with root length colonized by AMF, and that respiratory acclimation of roots to 7‐day cold‐temperature treatment was found in non‐AMF plants but not in AMF plants, equivalent to our conceptual Scenario D. In our study, *Ficus* and *Ochroma* showed an increase in respiration rate with increased percent AMF colonization. Acclimation was somewhat decreased by AMF colonization in *Luehea* roots, similarly to Atkin et al. (2009), whereas *Ochroma* roots showed the opposite of the results from Atkin et al. (2009) and the other two species showed no acclimation. In summary, *Ochroma* showed a trend for AMF‐stimulated acclimation, Scenario C, whereas *Luehea* showed a trend for AMF‐suppressed acclimation, Scenario D, while *Ficus* and *Tabebuia* showed no acclimation, Scenario A. Interestingly, no species showed acclimation independent of AMF, Scenario B. In contrast, Cheesman and Winter (2013a) found acclimation of leaf respiration to elevated nighttime temperature in both *Ficus* and *Ochroma*. Studies on whole plant and leaf respiratory acclimation across species also found high interspecific variation in acclimation, but mycorrhizal status of these plants was not assessed (Loveys et al. 2003; Cheesman and Winter 2013b). If systematic differences in AMF effects on thermal acclimation were to exist among different plant functional types, the complexity of hyperdiverse tropical forests could be reduced in predictive models of long‐term temperature effects on the tropical biosphere.

Surprisingly, only two of the four species showed an increase in respiration with colonization. Nottingham et al. (2010) found that 40% of root respiration in the tropical tree *Pseudobombax septenatum* was attributable to AMF extraradical mycelium. Peng et al. (1993) also found increased respiration in mycorrhizal versus nonmycorrhizal citrus roots of about 38%, but they found no difference in shoot respiration. For the two species that showed an increase in respiration with AMF colonization, this occurred in roots as well as shoots. This suggests that AMF can cause a whole plant up‐regulation of respiration rate, however this result should be interpreted with caution as shoot respiration was estimated as the difference between whole plant and root respiration.

Plant respiration rates can be limited by several factors, including the respiratory capacity, the demand for respiratory products, and the availability of respiratory substrates (mostly carbohydrates) (Atkin and Tjoelker 2003). Species tend to differ in which factor controls the respiration rates, with fast‐growing sun species more likely to be limited by substrate availability, and slow‐growing shade species being more constrained by respiratory capacity (Noguchi and Terashima 1997). AMF inoculation can in theory affect both the demand for respiratory products and the availability of respiratory substrates and as such, it has the potential to change the factor controlling the maximum rate of respiration. Atkin and Tjoelker (2003) identified two types of thermal acclimation; Type I acclimation, in which respiration is more strongly reduced at high temperatures than at low temperatures, which could be due to progressive substrate limitation at high temperature; and Type II acclimation, in which respiration is down‐regulated at all temperatures, thought to be associated with a reduction in respiratory capacity. If AMF inoculation affects the rate‐limiting factors of respiration, this is likely to also affect the acclimation response, both in terms of the type of acclimation, and in the capacity to achieve acclimation. Interestingly, even at 0% AMF inoculation, species differed in thermal acclimation, so even though species differed widely in the interactive effect of AMF inoculation and growth temperature, there are AMF‐independent species differences in acclimation capacity. Given the species richness of tropical forests and their importance in the global carbon cycle, more empirical data are required to assess the extent of interspecific variation in AMF effects on seedling response to warming.

We found that 70% of total CO_2_ was respired belowground regardless of percent AMF colonization. This ratio was nearly identical to that found in mycorrhizal citrus plants with high soil P, but was slightly higher than in nonmycorrhizal plants (64%) (Peng et al. 1993). Shoot R_25_ remained proportional to root R_25_ as percent AMF colonization increased, which indicates that plant metabolism increases in a balanced manner in both above‐ and belowground organs with mycorrhizal colonization. Interestingly, for species that showed acclimation, increased nighttime temperature caused a larger decrease in root R_25_ than shoot R_25_, indicating higher capacity for root respiratory acclimation.

Autotrophic respiration can be partitioned into growth and maintenance respiration. Maintenance respiration is the rate of respiration required for cellular maintenance and repair processes even when no growth occurs. This can be estimated as the y‐intercept of the regression of R_25_ against relative growth rate. Growth respiration, associated with biosynthesis, can be estimated from the slope of this RGR‐ R_25_ plot. In our study, the y‐intercepts of the regression of R_25_ versus RGR for mycorrhizal (11.1 *μ*mol kg^−1^ sec^−1^) and non‐mycorrhizal (7.8 *μ*mol kg^−1^ sec^−1^) plants indicate an increase in maintenance respiration with mycorrhizal colonization when all species data are pooled (Fig. 6; *F* = 24.2, *P* < 0.001). Growth respiration rates were not significantly higher in mycorrhizal plants than in non‐mycorrhizal plants. Mycorrhizal citrus plants were found to have higher growth and maintenance respiration even when AM and non‐AM plants had similar nutrition (Peng et al. 1993). They attributed higher growth respiration to higher root growth and construction costs (greater lipid content), while higher maintenance respiration was attributed to the higher root and fungal biomass and possible differences in ion uptake and transport.

We allowed seedlings to experience the natural diurnal variations in temperature over the course of the experiment, with warmed plants experiencing ~3°C temperature increase above ambient during the night. Warming night‐time only may have reduced the response to increased temperature in our study; however, respiration does not always acclimate to mean daily temperature (Atkin et al. 2000), and may, in fact, acclimate to nighttime temperature instead (Bruhn et al. 2007). Asymmetric day‐night warming is a realistic scenario for temperature regimes under future climate warming (Vose et al. 2005; IPCC 2007), and understanding how this type of warming will affect respiration and acclimation is crucial. In a future study it would be interesting to compare the effects of different types of warming scenarios on AMF and respiration.

### Growth and morphology

Relative growth rate increased with AMF colonization for two of the four species, indicating that even over the first few months of growth, mycorrhizal colonization can be beneficial to some species and not others. This could have important implications for early establishment and competition between species. AMF colonization also increased the shoot:root ratio of all species except *Ochroma*, indicating shoot growth is stimulated more strongly than root growth, and the increase in shoot respiration with AMF colonization is likely to result from respiration associated with this growth stimulation. Interestingly, AMF colonization increased respiration rate in both of the species that had no increase in RGR with AMF—*Ochroma* and *Ficus—*while in the two species with an increase in RGR with AMF colonization—*Luehea* and *Tabebuia*—there was no increase in R_25_ with AMF colonization. This may indicate species differences in the balance between the carbon cost and the growth benefit of the AM fungi, even though the increase in shoot *P* was similar across species.

We found that nighttime warming did not increase plant relative growth rate. In contrast, Cheesman and Winter (2013a) found increased seedling biomass of *Ochroma* and *Ficus* under elevated nighttime temperature. Additionally, Cheesman and Winter (2013a) found an increase in shoot to root ratio and leaf area in warmed *Ochroma* and *Ficus* plants, while in our study allocation to above‐ and belowground portions were similar at elevated and ambient temperature. This difference may have been due to greater nutrient limitation in our study with unfertilized plants, which prevented temperature‐stimulated growth. On the other hand, we found that specific leaf area, leaf area ratio, and shoot to root ratio increased with mycorrhizal colonization, indicating increased allocation to photosynthetic tissues.

It is widely recognized that AMF play an important role in tropical forest productivity and diversity (Alexander et al. 1992). Given the high abundance of mycorrhizal plants in tropical forests and the importance of tropical forests in regulating global climate, it is critical that we gain a better understanding of the effect of AM colonization on plant respiration rate and acclimation potential. Here, we show that AM colonization increases respiration rate of plant roots and shoots, and that AM colonization has the potential to increase respiratory acclimation in some species, but not in others. High variability in acclimation across species and mycorrhizal treatments caution the potential errors in carbon flux models that ignore these factors, strongly suggesting the importance of continuing to investigate the mechanisms of this variability.

## Conflict of Interest

None declared.

## Supporting information


**Table S1**. Results of linear model used to test for differences in percent AMF colonization for seedlings with nonzero colonization between warmed and ambient grown plants and with plant age at harvest.
**Table S2**. Comparison of nighttime soil and air temperature (°C) in the warmed and ambient treatments.
**Figure S1**. Representative temperatures (22–23 July 2012) measured every 10 min with iButton data loggers suspended near the leaves or placed on the soil surface of ambient or warmed seedlings grown in the greenhouse with heat rope.
**Figure S2**. Representative temperatures (27 July–2 August 2012) of warmed and ambient grown plants in 2 types of warming treatment, either grown in the greenhouse with heat rope (Greenhouse) or in an open top chamber (Chamber).
**Figure S3**. Mean root, shoot, and whole plant respiration rate measured at 25°C (R_25_) of *Castilla* seedlings grown at ambient or warmed nighttime temperature.
**Figure S4**. Linear regression of the ratio of root R_25_ to shoot R_25_ versus AM colonization.
