# Supplementary material for: Influence of arbuscular mycorrhizal colonization on whole‐plant respiration and thermal acclimation of tropical tree seedlings
Source: Ecol Evol. 2016 Jan 18;6(3):859–70. doi: 10.1002/ece3.1952 (PMC4739557; doi:10.1002/ece3.1952)

Appendix

Table S1: Results of linear model used to test for differences in percent AMF colonization for seedlings with nonzero colonization between warmed and ambient grown plants and with plant age at harvest.

|  |  | *Ficus* |  |  | *Luehea* |  |  | *Ochroma* |  |  | *Tabebuia* |  |
| --- | --- | --- | --- | --- | --- | --- | --- | --- | --- | --- | --- | --- |
|  | df | F | P |  | F | P |  | F | P |  | F | P |
| Temperature | 1 | 0.1 | 0.81 |  | <0.1 | 0.94 |  | 0.8 | 0.39 |  | 2.0 | 0.19 |
| Plant age | 1 | 0.3 | 0.62 |  | **7.8** | **0.02** |  | 0.2 | 0.64 |  | 1.6 | 0.23 |

Table S2. Comparison of nighttime soil and air temperature (°C) in the warmed and ambient treatments.

|  | Warmed soil | Ambient soil | Warmed shoot | Ambient shoot |
| --- | --- | --- | --- | --- |
| Heat rope | 27.76 (0.97) | 25.48 (1.50) | 27.60 (1.12) | 24.47 (1.00) |
| Open top chamber | 27.32 (0.87) | 25.70 (1.09) | 28.61 (1.31) | 24.75 (1.43) |

Figure S1. Representative temperatures (22-23 July 2012) measured every 10 minutes with iButton data loggers suspended near the leaves or placed on the soil surface of ambient or warmed seedlings grown in the greenhouse with heat rope.

Figure S2. Representative temperatures (27 July - 2 August 2012) of warmed and ambient grown plants in 2 types of warming treatment, either grown in the greenhouse with heat rope (Greenhouse) or in an open top chamber (Chamber). Temperatures were recorded with iButton data loggers suspended near the leaves.


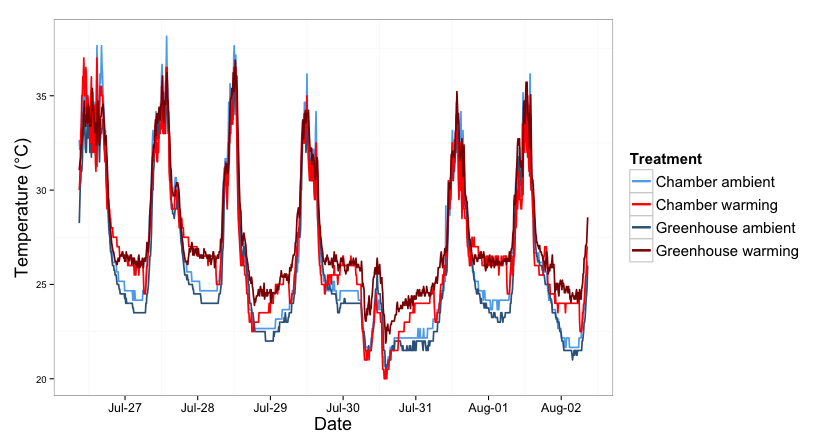


Figure S3. Mean root, shoot, and whole plant respiration rate measured at 25 °C (R_25_) of *Castilla* seedlings grown at ambient or warmed nighttime temperature. Error bars indicate standard error.


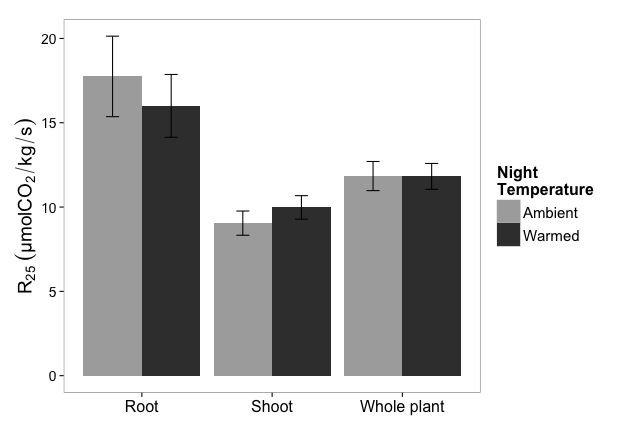


Figure S4. Linear regression of the ratio of root R_25_ to shoot R_25_ versus AM colonization.


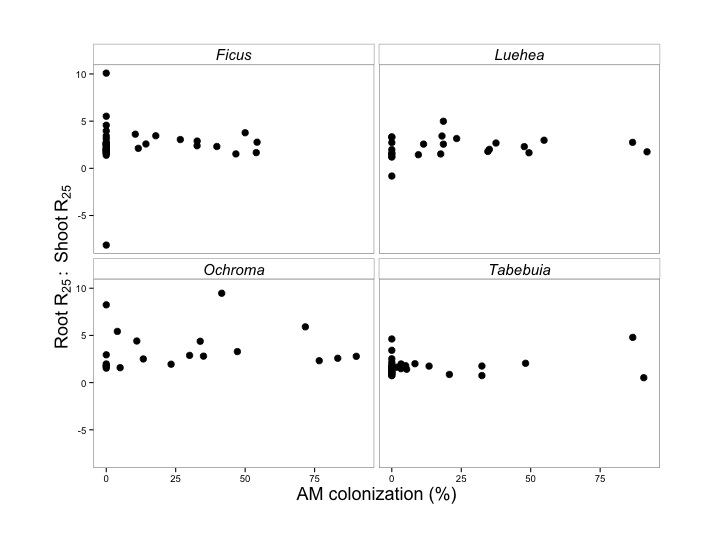

Supplement: Supplementary file 1 — Table S1. Results of linear model used to test for differences in percent AMF colonization for seedlings with nonzero colonization between warmed and ambient grown plants and with plant age at harvest. Table S2. Comparison of nighttime soil and air temperature (°C) in the warmed and ambient treatments. Figure S1. Representative temperatures (22–23 July 2012) measured every 10 min with iButton data loggers suspended near the leaves or placed on the soil surface of ambient or warmed seedlings grown in the greenhouse with heat rope. Figure S2. Representative temperatures (27 July–2 August 2012) of warmed and ambient grown plants in 2 types of warming treatment, either grown in the greenhouse with heat rope (Greenhouse) or in an open top chamber (Chamber). Figure S3. Mean root, shoot, and whole plant respiration rate measured at 25°C (R25) of Castilla seedlings grown at ambient or warmed nighttime temperature. Figure S4. Linear regression of the ratio of root R25 to shoot R25 versus AM colonization. [file ECE3-6-859-s001.docx]
